# Mild-photothermal and nanocatalytic therapy for obesity and associated diseases

**DOI:** 10.7150/thno.99948

**Published:** 2024-09-03

**Authors:** Lewen Zheng, Aung Than, Ping Zan, Dongsheng Li, Zheye Zhang, Melvin Khee Shing Leow, Peng Chen

**Affiliations:** 1 School of Chemistry, Chemical Engineering and Biotechnology, Nanyang Technological University, 637457, Singapore.; 2 College of Materials and Chemical Engineering, Key Laboratory of Inorganic Nonmetallic Crystalline and Energy Conversion Materials, China Three Gorges University, Yichang, 443002, P. R. China.; 3 Department of Endocrinology, Tan Tock Seng Hospital, 308433, Singapore.; 4 Lee Kong Chian School of Medicine, Institute for Digital Molecular Analytics and Science, Nanyang Technological University, 636921, Singapore.; 5 Skin Research Institute of Singapore, 308232, Singapore.

**Keywords:** photothermal therapy, nanocatalytic therapy, transdermal delivery, metabolism, obesity, diabetes

## Abstract

**Background:** Current anti-obesity medications suffer from limited efficacy and side-effects because they act indirectly on either the central nervous system or gastrointestinal system. Herein, this work aims to introduce a transdermal photothermal and nanocatalytic therapy enabled by Prussian blue nanoparticles, which directly act on obese subcutaneous white adipose tissue (sWAT) to induce its beneficial remodeling including stimulation of browning, lipolysis, secretion of adiponectin, as well as reduction of oxidative stress, hypoxia, and inflammation.

**Methods:** Prussian blue nanoparticles were synthesized and incorporated into silk fibroin hydrogel for sustained retention. The efficacy of mild photothermal (808 nm, 0.4 W/cm^2^, 5 min) and nanocatalytic therapy (mPTT-NCT) was assessed both *in vitro* (3T3-L1 adipocytes) and *in vivo* (obese mice). The underlying signaling pathways are carefully revealed. Additionally, biosafety studies were conducted to further validate the potential of this therapy for practical application.

**Results:** On 3T3-L1 adipocytes, mPTT-NCT was able to induce browning, enhance lipolysis, and alleviate oxidative stress. On obese mice model, the synergistic treatment led to not only large mass reduction of the targeted sWAT (53.95%) but also significant improvement of whole-body metabolism as evidenced by the substantial decrease of visceral fat (65.37%), body weight (9.78%), hyperlipidemia, and systemic inflammation, as well as total relief of type 2 diabetes.

**Conclusions:** By directly targeting obese sWAT to induce its beneficial remodeling, this synergistic therapy leads to significant improvements in whole-body metabolism and the alleviation of obesity-related conditions, including type 2 diabetes. The elucidation of underlying signaling pathways provides fundamental insights and shall inspire new strategies to combat obesity and its associated diseases.

## Introduction

White adipose tissue (WAT) stores excess energy in the form of triglycerides and regulates whole-body metabolism through the secretion of a variety of adipokines 1-4. But obese WAT is characterized by various pathological conditions including oxidative stress, chronic inflammation, hypoxia, over-release of free fatty acids and proinflammatory factors, and altered secretion of adipokines, which increase the risk of developing various critical diseases, such as type 2 diabetes, cardiovascular diseases, and some cancers 5-7. The current medications for obesity (including the recently commercialized GLP-1 agonists) act indirectly on the central nervous system or gastrointestinal system to suppress appetite, gastric emptying, or fat adsorption. Therefore, they suffer from poor effectiveness or side effects 8. Thus, it is desirable to directly target the root of the problems by inducing ameliorative remodeling of obese WAT. For example, transforming white adipocytes into energy-burning brown-like (beige) adipocytes (a process called browning) has been shown to be a promising strategy 9-12. Recently, our group and another team have shown that localized hyperthermia induced by mild photothermal thermal therapy (mPTT) can promote browning of WAT whereby leading to reduction of fat mass, hyperlipidemia, and hyperglycemia 13, 14. We demonstrated that activation of transient receptor potential vanilloid 1 (TRPV1) is the key whereas the other group demonstrated the importance of heat shock factor 1 (HSF1). But the browning strategy can be compromised by the pathological conditions such as oxidative stress, hypoxia and chronic inflammation in obese WAT 15, 16. In addition, frequent photothermal treatment could cause inflammation.

**Figure 1:**
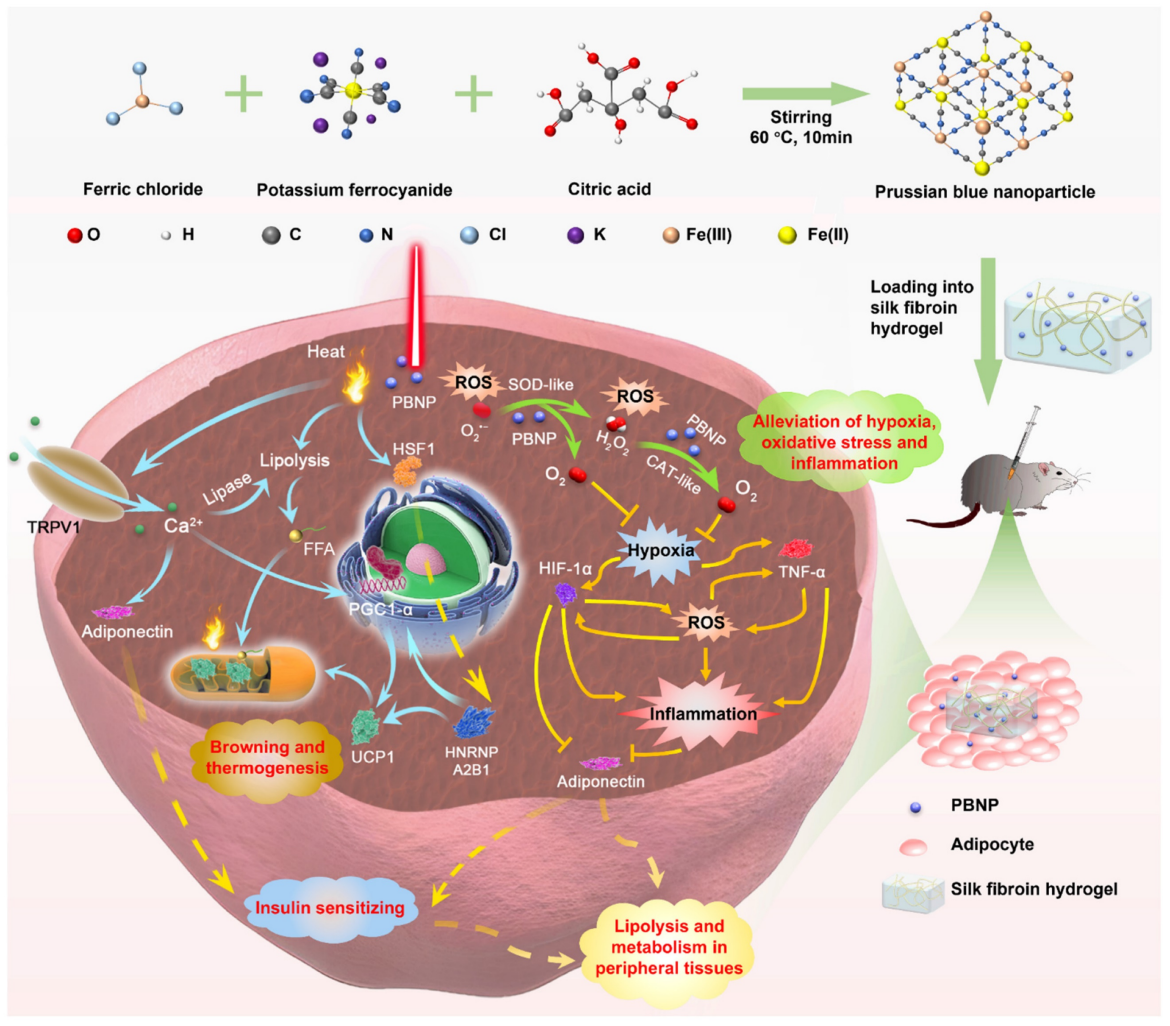
Schematic illustration of the synthesis of PBNPs, application of mild-photothermal and nanocatalytic therapy, and the underlying mechanisms.

The abovementioned pathological conditions intriguingly couple with each other, forming vicious positive feedback loops 17. They are the root causes to various obesity-associated diseases. We conceive that beneficial remodeling of obese WAT by relieving oxidative stress, hypoxia and inflammation can also offer anti-obesity effect and synergize with photothermal therapy. In this work (**Figure 1**), Prussian blue nanoparticles (PBNPs) were synthesized, which serve simultaneously as a photothermal agent and a nanozyme mimicking superoxide dismutase (SOD) and catalase (CAT) to enable photothermally induced browning, scavenging of reactive oxygen species (ROS), and O_2_ production. Biocompatible silk fibroin hydrogel was devised to encapsulate PBNPs for readily transdermal injection and lasting retention in obese subcutaneous WAT (sWAT). The induced adipose remodeling not only largely reduces the mass of the targeted sWAT, but also significantly improves the whole-body metabolism as evidenced by total relief of type 2 diabetes and reduction of visceral fat, serum cholesterol, systemic oxidative stress and inflammation. Moreover, it was revealed that mPTT-triggered browning is achieved through both TRPV1 and HSF1 pathways, and nanocatalytic therapy (NCT) enabled by PBNPs breaks the vicious coupling between oxidative stress, hypoxia and inflammation (**Figure 1**). The intriguing and interconnected signaling pathways underlying the synergistic mPTT-NCT are carefully revealed, providing new insights to inspire novel strategies to combat obesity and associated diseases.

## Materials and methods

### Materials

Iron(III) chloride, potassium hexacyanoferrate(II) trihydrate, citric acid, sodium carbonate, lithium bromide, nitroblue tetrazolium (NBT), riboflavin, 1-ethyl-3-(3-(dimethylamino)propyl) carbodiimide hydrochloride (EDC) and N-hydroxysulfosuccinimide (NHS) were obtained from Sigma-Aldrich. Cy5-amine was purchased from Lumiprobe. Mulberry (Bombyx) silk cocoons were purchased from Woolery.

### Synthesis, Functionalization, and Characterization of Prussian Blue Nanoparticles (PBNPs)

To a 20 mL solution of 1 mM FeCl_3_, 0.5 mmol (96 mg) of citric acid was added. Afterwards, a 20 mL solution of 1 mM K_4_[Fe(CN)_6_] containing the same amount of citric acid was added. The resulting mixture was stirred at 60 °C for 10 min to produce a bright blue aqueous dispersion of PBNPs, followed by dialysis (MWCO: 5 kDa) for 24 h to remove the remaining reactants. The absorption spectrum, size and morphology of PBNPs were analyzed by spectrophotometer (UV-1800, Shimadzu), dynamic light scattering (Malvern Nano-ZS Zeta Sizer) and transmission electron microscope (JEM 3010 TEM), respectively. Fourier transform infrared (FTIR) analysis was performed on an infrared spectrometer (Nicolet iS50). X-ray photoelectron spectroscopy (XPS) measurement was conducted with an XPS spectrometer (Kratos AXIS Supra). Energy Dispersive X-ray elemental mapping was performed by Oxford X-Max Energy Dispersive X-ray Analyser. To conjugate fluorescent dye Cy5 onto PBNPs (Cy5-PBNP), the carboxyl groups on the surface of PBNPs were first activated. Specifically, 33.6 μM 1-ethyl-3-(3-(dimethylamino)propyl) carbodiimide hydrochloride (EDC) and 33.6 μM N-hydroxysulfosuccinimide (NHS) were added into 10 mL PBNPs solution and stirred for 30 min. Then, 10 μL of Cy5-amine (25 mg/mL) was introduced, followed by stirring for 12 h and dialysis (MWCO: 5 kDa) for 24 h to remove unbound Cy5 molecules.

### Superoxide Dismutase (SOD) Mimetic Activity of PBNPs

The superoxide anion (O_2_^•-^) scavenging activity of PBNPs was assessed by the NitroBlue Tetrazolium/Riboflavin (NBT/RF) method. The reaction mixture in PBS contained EDTA (0.1 mM), riboflavin (1 mM), NBT (50 µM) and various concentrations of PBNPs (25, 50, and 100 µg/mL) were prepared and uniformly illuminated by white light (Arc Lamp, Newport) for 2 min. The optical absorbance at 540 nm was measured before and after the illumination.

### Catalase (CAT) Mimetic Activity of PBNPs

In a PBS solution, H_2_O_2_ (50 mM) was mixed with various concentrations of PBNPs (0, 25, 50, and 100 µg/mL). The dissolved oxygen levels were then measured using a portable dissolved oxygen meter (CLEAN Instruments Co.,Ltd, China).

### Cells and Animals

3T3-L1 cells, purchased from Chemoscience Pte Ltd (Singapore), were cultured in a DMEM medium supplemented with 10% calf bovine serum (CBS) and maintained in a 5% CO_2_ atmosphere at 37°C. Male C57BL/6J mice, aged 7 to 8 weeks with an average body weight of 22.66 g, were procured from the Animal Research Facility of Nanyang Technological University and housed in a controlled temperature (23.5 °C) and a 12-hour light/dark cycle. They had free access to food and water. All animal experiments were conducted in accordance with ethical guidelines approved by the Institutional Animal Care and Use Committee of Nanyang Technological University (NTU-IACUC, A23038).

### Preparation and Characterization of Silk Fibroin Gel

Briefly, 5 g of silk cocoons were finely chopped and treated with 0.02 M Na_2_CO_3_ solution (2 L) at boiling temperature for 30 min to remove sericin. The degummed silk fibers were then washed thoroughly with deionized water for 30 min (three times) to remove residual Na_2_CO_3_ and sericin. After air drying overnight, the washed silk fibers were weighed and dissolved in 9.3 M LiBr solution (20% w/v) at 60 ℃ for 4 h. Subsequently, the solution was dialyzed against deionized water using a dialysis bag (MWCO 3500D) at 4 ℃ for 3 days to remove any remaining small molecules. Finally, the silk fibroin solution was obtained by centrifuging twice at 12000 rpm for 20 min. The concentration of the solution was determined by measuring the mass before and after complete drying. To prepare PBNP-loaded silk gel, a defined amount of PBNPs was dispersed in a 1% w/v solution of silk fibroin, followed by immersion in an ultrasonic bath for 30s to stimulate formation of the β-sheet structure of silk fibroin for gelation. The resulting mixture was then transferred into a 37 ℃ oven for overnight gelation. Similarly, silk gel without PBNPs was also prepared. The viscosity of the gel was analyzed by Anton Paar MCR 102e Rheometer.

### The *In Vitro* and *In Vivo* Release Profile of PBNPs from Silk Gel

To study the *in vitro* release, PBNPs loaded silk gel was immersed in PBS solution. At the designated time points, 1 mL sample was collected and replaced with the same volume of fresh PBS. The amount of PBNPs released was quantified by a predetermined calibration equation using a UV spectrophotometer. To investigate the *in vivo* release profile, Cy5-PBNP loaded silk gel was injected subcutaneously into the inguinal fat of mice, and the mice were observed at specific time intervals using an *in vivo* imaging system (IVIS Spectrum) with excitation at 640 nm and emission at 680 nm. The *in vivo* release profile of Cy5-PBNPs was determined by the decrease in fluorescence intensity.

### Photothermal Performance of PBNP-Gel

Various concentrations of PBNPs (0, 25, 50, and 100 µg/mL) in 1% w/v silk gel were prepared and exposed to 808 nm laser irradiation at different power densities (0.2, 0.4, and 0.6 W/cm^2^) for 5 min. The temperature of the sample was monitored in real-time using a FLIR thermal camera. To assess the photothermal stability of PBNP-gel, a laser on/off cycle experiment was conducted. In each cycle, the sample was irradiated at 0.4 W/cm^2^ for 300 s, followed by cooling down naturally with laser irradiation.

### Evaluation of the Photothermal Effect of PBNP-gel *In Vivo*

8 mice were randomly divided into two groups: silk + 808 nm and silk + 808 nm + PBNPs. 50 μL of silk gel, with or without PBNPs (50 µg/mL), was injected into the inguinal fat of the mice. Subsequently, the mice were exposed to 808 nm laser irradiation at 0.4 W/cm^2^ for 5 min, while real-time temperature changes and images of the inguinal region were recorded by a FLIR thermal camera (T420, FLIR).

### *In Vivo* Treatment

Mice were fed with a high-fat diet (58Y1, 60% kcal provided by fat, Testdiet) for 4 weeks. Then the obese mice with an average body weight of 37.1 g were randomly divided into 5 groups with a continuous high-fat diet feeding for different treatments. The inguinal regions of all mice were depilated for the treatment. Under anesthesia, mice were subjected to subcutaneous injection of silk gel (1% w/v, 3 mL/kg; once a week) as control, or silk gel (once a week) followed by NIR laser irradiation (808 nm, 0.4 W/cm^2^, 5 min; 14 consecutive days), or silk gel containing PBNPs (0.15 mg/kg; once a week), or silk gel containing PBNPs (0.15 mg/kg; once a week) followed by NIR laser irradiation, or treated with an intraperitoneal injection of heat shock factor 1 inhibitor: DTHIB (5 mg/kg, Axon Medchem; 5 consecutive days) followed by subcutaneous injection of silk gel containing PBNPs (0.15 mg/kg; once a week) and NIR laser irradiation. For the glucose tolerance test on day 12 of treatment, mice were fasted overnight, followed by intraperitoneal injection of glucose (1 g/kg in PBS). The blood glucose levels in blood samples from the tail vein were measured at different time points with a glucometer (Accu-Chek Performa, Roche). On day 14, the mice's body surface temperature was recorded when mice were fully awake using the infrared camera. The enzyme-linked immunosorbent assay (ELISA) kits (Abcam or Thermo Fisher Scientific) and colorimetric quantification kits (Sigma-Aldrich) were used for serological analysis. Mice were then euthanized, and adipose tissues (IgWAT, EpiWAT, interscapular BAT) and major organs (liver, kidneys, spleen, lung, heart) were excised, weighed, and fixed for H&E staining and immunohistological analyses.

### Statistical Analyses

Quantitative data is presented as mean ± standard deviation (s.d.) with error bars on all graphs. Statistical analyses were performed using GraphPad Prism 8, with one-way analysis of variance (ANOVA) used for data analysis. Statistical significance was set at P < 0.05, denoted by asterisks (*P < 0.05, **P < 0.01, ***P < 0.001).

## Results

### Synthesis and Characterization of PBNPs and PBNP-Gel

**Figure 2:**
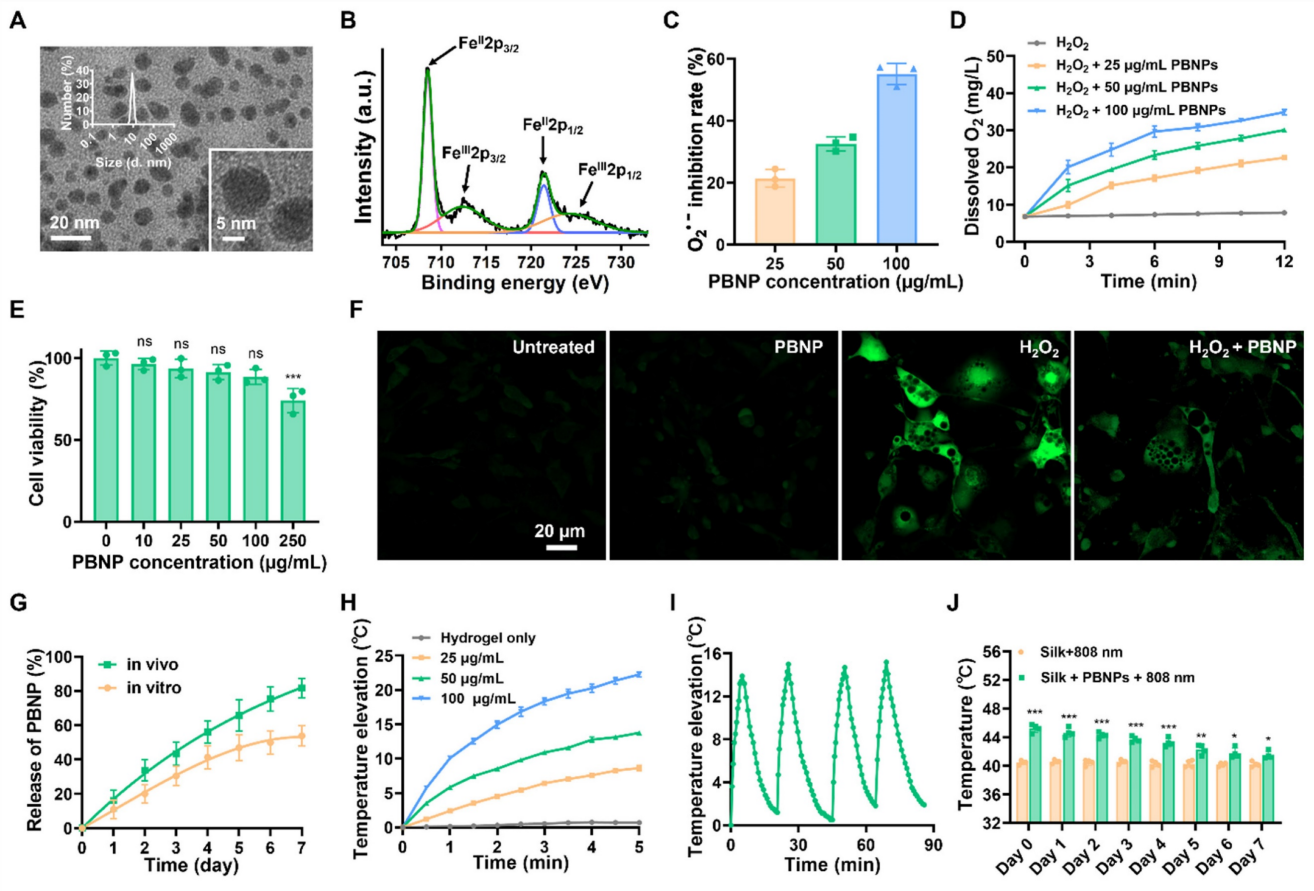
Characterization of PBNP and PBNP-gel. **(A)** TEM images of PBNPs and size distribution obtained d by DLS. **(B)** Fe 2p XPS spectra of PBNP. **(C)** O_2_^•-^ scavenging activity of PBNP at various concentrations in PBS. **(D)** Oxygen generation from H_2_O_2_ with different PBNP concentrations in PBS. **(E)** Viability of 3T3-L1 adipocytes after incubation with PBNPs of different concentrations for 24 h. Data represents mean ± s.d. (n = 3). One-way ANOVA: ns = not significant; ***P < 0.001 versus control. **(F)** Fluorescent images of ROS levels in 3T3-L1 adipocytes using DCFH-DA dye as the reporter. **(G)** *In vitro* and *in vivo* release profile of PBNPs from silk gel. **(H)** Temperature changes of silk hydrogel with different PBNP concentrations under 5 min of 808 nm NIR irradiation with a power density of 0.4 W/cm^2^. **(I)** Photothermal stability of PBNPs (50 µg/mL) in silk hydrogel subjecting to four NIR irradiation cycles. **(J)** Surface temperature at the inguinal region after 5 min NIR irradiation on different days after subcutaneous injection of PBNP-gel. Data represents mean ± s.d. (n = 4). One-way ANOVA: *P < 0.05, **P < 0.01, ***P < 0.001 versus control.

Prussian blue nanoparticles (PBNPs) were synthesized through a facile solvothermal reaction between ferric chloride and potassium ferrocyanide in the presence of citric acid as the surface capping agent to prevent aggregation. (**Figure 1**). The transmission electron microscopy (TEM) image and dynamic scattering light (DLS) measurement reveal that the spherical PBNPs have an average diameter of ~9 nm (**Figure 2A**). Such a small size is favorable for cellular uptake and being cleared or metabolized by the body. PBNPs are incorporated with a redox couple, i.e., Fe(III) and Fe(II) atoms. The transition between them endows the NP with nanocatalytic ability. As shown in **Figure 2B**, the X-ray photoelectron spectrometry (XPS) spectrum of Fe2p presents the characteristic peaks of Fe^II^2p_3/2_ and Fe^II^2p_1/2_ at the binding energies of 708.5 eV and 721.6 eV, and the peaks of Fe^III^2p_3/2_ and Fe^III^2p_1/2_ at 712.5 eV and 725.7 eV, confirming the coexistence of Fe(II) and Fe(III). In addition, as shown in the Fourier transform infrared (FTIR) spectrum (**Figure S1**), PBNPs exhibit a transmittance peak at 2089 cm^-1^, which is the characteristic stretching frequency of C≡N bond. Furthermore, the peak at 599 cm^-1^ indicates the existence of Fe^2+^-C≡N-Fe^3+^ bonds in the PBNP framework 18. Further, energy Dispersive X-ray elemental mapping also demonstrates the uniform presence of C, N and Fe elements in PBNPs (**Figure S2**). These observations confirm the successful synthesis of PBNPs.

Superoxide radical (O_2_^•-^) and H_2_O_2_ are the major ROS inevitably generated from cellular metabolism and are over-abundant in obese fat 19. Mimicking superoxide dismutase (SOD), PBNP decorated with both Fe(II) and Fe(III) is able to catalyze O_2_^•-^ into H_2_O_2_ and O_2_ in a two-step disproportionation reaction. Firstly, Fe(II) transits to Fe(III) by donating an electron to O_2_^•-^, leading to the production of H_2_O_2_; secondly, Fe(III) is reduced to Fe(II) by accepting electrons from another O_2_^•-^, leading to generation of O_2_ 20. To investigate the SOD-like activity of PBNP, NitroBlue Tetrazolium/Riboflavin (NBT/RF) method was applied to report O_2_^•-^. Specifically, under light irradiation, riboflavin produces O_2_^•-^ to reduce NBT to formazan, which absorbs light at 540 nm. As shown in **Figure 2C**, PBNP eliminates O_2_^•-^ in a dose-dependent manner. Mimicking catalase (CAT), PBNP catalyzes H_2_O_2_ into H_2_O and O_2_ in a similar two-step disproportionation reaction 20. Its CAT-like activity was evidenced by the bubble generation and increase of dissolved O_2_ in a PBNP concentration dependent manner (**Figure 2D and Figure S3**). By monitoring the size and characteristic UV absorbance, it was concluded that PBNP could stably catalyze O_2_^•-^ and H_2_O_2_ without degradation (**Figure S4 and S5**). Altogether, PBNP shall be able to sustainably reduce the oxidative stress and hypoxia in obese fat through its dual nanocatalytic activities. Conjugating PBNPs with the fluorescence dye Cy5, confocal imaging revealed that PBNPs can be readily uptaken by 3T3-L1 adipocytes (**Figure S6**). PBNPs exhibited negligible cytotoxicity to 3T3-L1 adipocytes in concentrations up to 100 µg/mL (**Figure 2E**). In addition, as reported by DCFH-DA (a ROS-sensitive fluorescence dye), H_2_O_2_ (500 µM) induced intracellular oxidative stress was largely relieved by PBNPs (100 µg/mL) (**Figure 2F**).

Injectable silk fibroin hydrogel is an appealing delivery vehicle for therapeutics due to its biocompatibility, absence of immune responses, biodegradability, and tunable properties 21-24. By fine-tuning the fibroin concentration and crosslinking conditions, the synthesized hydrogel simultaneously achieves good injectability and sustainable drug release of therapeutics. **Figure S7** shows that at a shear rate of 1 s^-1^, 50 µg/mL PBNP-loaded 1% fibroin gel (PBNP-gel) exhibited low viscosity at both room temperature (0.49 Pa·s) and body temperature (0.45 Pa·s), allowing for easy injection into the body through a fine insulin needle. After immersing PBNP-gel in PBS for 7 days, 53.6% of PBNPs were released (**Figure 2G**). Further, fluorescence dye Cy5 was conjugated onto PBNP to track the nanoparticles after subcutaneous injection into the inguinal fat (IgWAT) of mice. Based on the decrease of fluorescence intensity at the injection site, it was determined that 81.25% of Cy5-PBNPs were released in 7 days (**Figure 2G and Figure S8**). The *in vivo* release is faster than *in vitro* release presumably due to the existence of proteases.

To assess the photothermal performance, PBNP-gel was subjected to NIR laser irradiation (808 nm, 0.4 W/cm^2^) for 5 min. The temperature increase of PBNP-gel is laser power (**Figure S9**) and PBNP concentration dependent (**Figure 2H**). At 50 µg/mL, an increase of 13.9 ℃ was attained in 5 min with a photothermal conversion efficiency of 26.9% and high photo-stability (**Figure 2I**). In addition, the photothermal effect does not affect the release profile of PBNPs from silk gel (**Figure S10**). To evaluate the *in vivo* performance, PBNP-gel was injected into IgWAT (~2.5 mm below the skin) and an infrared thermal camera was used to monitor the surface temperature during laser irradiation. Right after injection, the surface temperature increased to 45.1 ℃ with 5 min laser irradiation, which is 5.5 ℃ higher than laser irradiation alone. Note that, with the injected PBNP-gel, the actual adipose temperature is higher than the surface temperature whereas it is opposite with laser irradiation alone. The laser-induced hyperthermia gradually decreased over time due to the diffusion-away of PBNPs. But even at day 7, photothermal induced surface temperature is still 2.3 ℃ higher than laser irradiation alone (**Figure 2J**). These observations suggest that PBNP-gel allows continuous treatment for 1 week.

### *In Vitro* Effects of mPTT-NCT on 3T3-L1 Adipocytes

**Figure 3:**
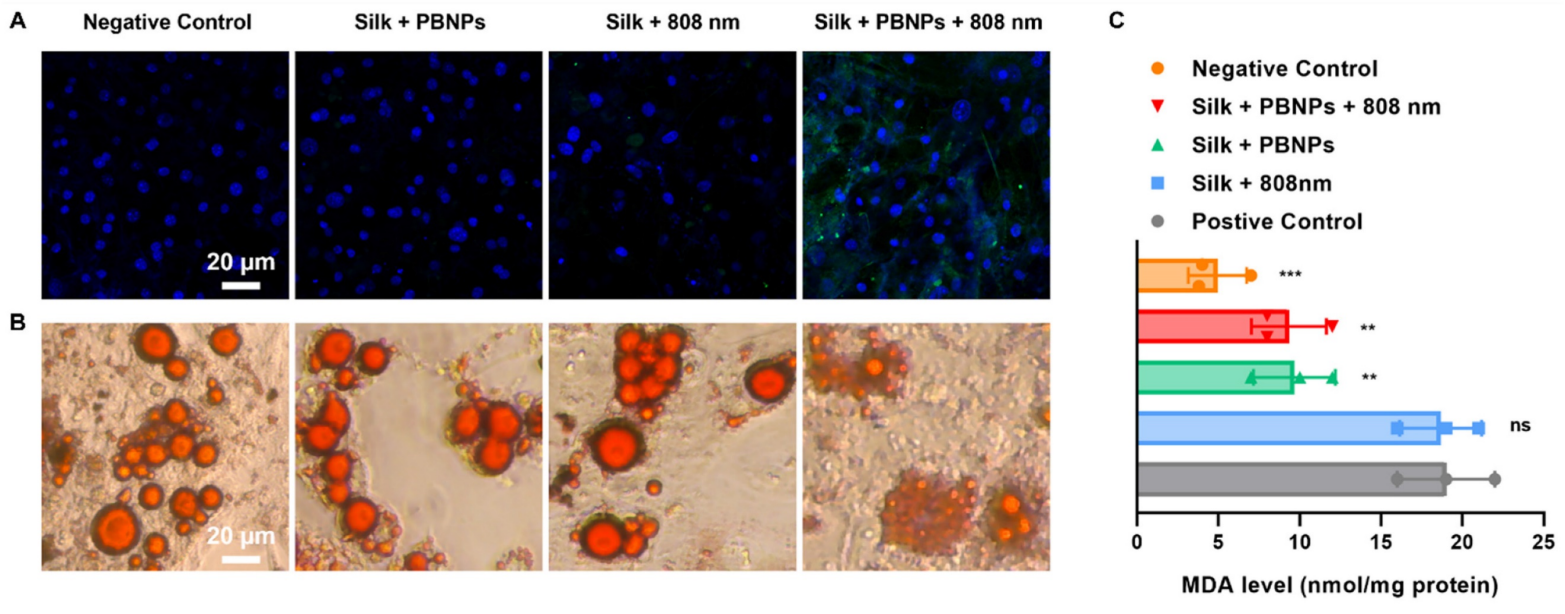
*In vitro* treatment efficacy on 3T3-L1 adipocytes. **(A)** UCP1-labeled (green) immunofluorescence images with nuclei being stained blue. **(B)** Oil Red O staining of intracellular lipid droplets. **(C)** Cellular malondialdehyde (MDA) levels after different treatments. Positive control: incubate cells with 500 µM H_2_O_2_ for 3 h. Negative control: without any treatment. Data represents mean ± s.d. (n = 3). One-way ANOVA: n.s. = not significant; **P < 0.01; ***P < 0.001 versus positive control.

In contrast to other treatments without PBNP or laser irradiation, mPTT-NCT treatment notably increased the expression of uncoupling protein 1 (UCP1), a brown-adipocyte specific mitochondrial protein responsible for thermogenic respiration (**Figure 3A**). This implies that mPTT-NCT induced the browning of white adipocytes. Additionally, lipolysis was promoted by mPTT-NCT as evidenced by the breakdown of large intracellular lipid droplets into small ones (**Figure 3B**). Moreover, mPTT-NCT largely mitigated H_2_O_2_-induced oxidative stress in adipocytes, indicated by the obvious decrease of cellular malondialdehyde (MDA) (**Figure 3C**). In summary, these findings underscore the ability of mPTT-NCT to induce browning, enhance lipolysis, and alleviate oxidative stress in adipocytes.

### *In Vivo* Effects of mPTT-NCT on Obesity

**Figure 4:**
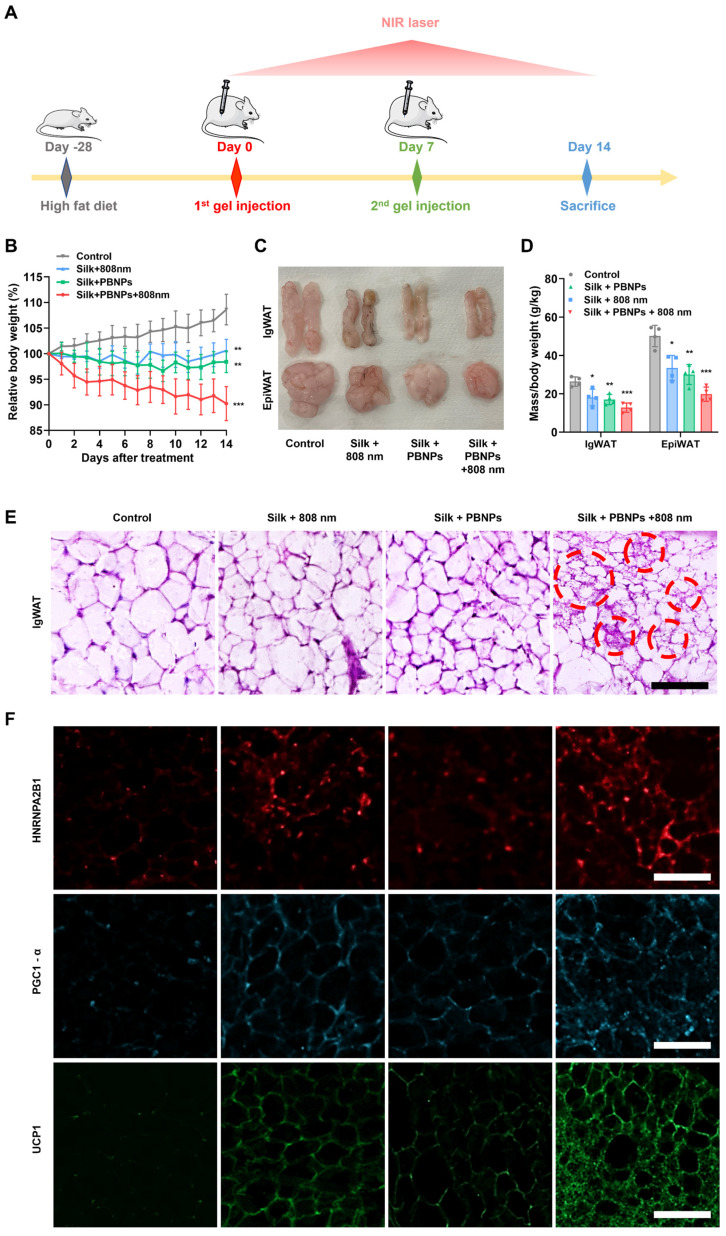
*In vivo* treatment efficacy on obese mice.** (A)** Protocol illustration. **(B)** Relative body weight changes over the course of treatment. **(C)** Photographs and **(D)** relative fat masses of IgWAT and EpiWAT after the treatment. Data represents mean ± s.d. (n = 4). One-way ANOVA: ns = not significant; *P < 0.05, **P < 0.01, ***P < 0.001 versus control. **(E)** H&E staining images of IgWAT. Red circle indicates a representative brown-like adipocyte with multilocular morphology. **(F)** HNRNPA2B1-labeled (red), PGC1-α-labeled (blue) and UCP1-labeled (green) immunofluorescence images of IgWATs after different treatments. Scale bar = 100 μm.

Mice with an initial average body weight of 22.6 g were fed with a high-fat diet (HFD) for 4 weeks. The substantial weight gain to an average of 37.1 g (64.16% increase) and developed hyperglycemia and hyperinsulinemia (**Figure S11**) confirmed the successful establishment of obese mouse models 25, 26. The obese mice were divided into 4 groups for different treatments (silk gel as control, silk gel + NIR laser, PBNP-gel, PBNP-gel + NIR laser). As illustrated in **Figure 4A**, in the treatment course of 2 weeks, silk gel (3 mL/kg) with or without PBNP (0.15 mg/kg) was subcutaneously injected into the sWAT underneath the inguinal regions (IgWAT) at day 0 and day 7, and for some groups, 808 nm laser irradiation (0.4 W/cm^2^) was applied for 5 min every day.

For the control group, the body weight of obese mice continued to gain 9.61%, whereas PBNP-gel with NIR laser irradiation offered outstanding anti-obesity effect as evidenced by a weight loss of 9.78% (**Figure 4B and Figure S12**). Consistent with the previous observation 13, laser irradiation without the photothermal agent also suppressed the obesity development (0.58% weight gain) because photothermal effects can be directly induced in IgWAT through the thin mouse skin (0.55 mm thick). But PBNPs are critical in order to achieve better therapeutic effects and apply such transdermal therapy on thick human skin (up to 4 mm thick). In addition, PBNP-gel without NIR laser irradiation led to a 1.59% weight reduction, demonstrating the anti-obesity effect originated from the antioxidant and O_2_-producing properties of PBNPs. Noteworthy, the food intake was similar for all mouse groups, suggesting that the observed therapeutic outcomes were not caused by disturbing the behavior (**Figure S13**).

Furthermore, subcutaneous IgWAT and visceral epididymal white adipose tissue (EpiWAT) shrunk most significantly after PBNP-gel with NIR laser irradiation treatment (53.95% less for IgWAT; 65.37% less for EpiWAT, **Figure 4C and 4D**). A large reduction of visceral fat implies that local subcutaneous treatment on IgWAT exerts systemic anti-obesity effects through endocrine signaling pathways. Browning of the targeted IgWAT was evidenced by elevated body temperature at the inguinal regions (**Figure S14**). Moreover, thermogenic multilocular brown-like adipocytes with a much smaller size due to enhanced lipolysis appeared in IgWAT after the mPTT-NCT (**Figure 4E and Figure S15**).

### Revealing the Signaling Pathways Underlying mPTT

**Figure 5:**
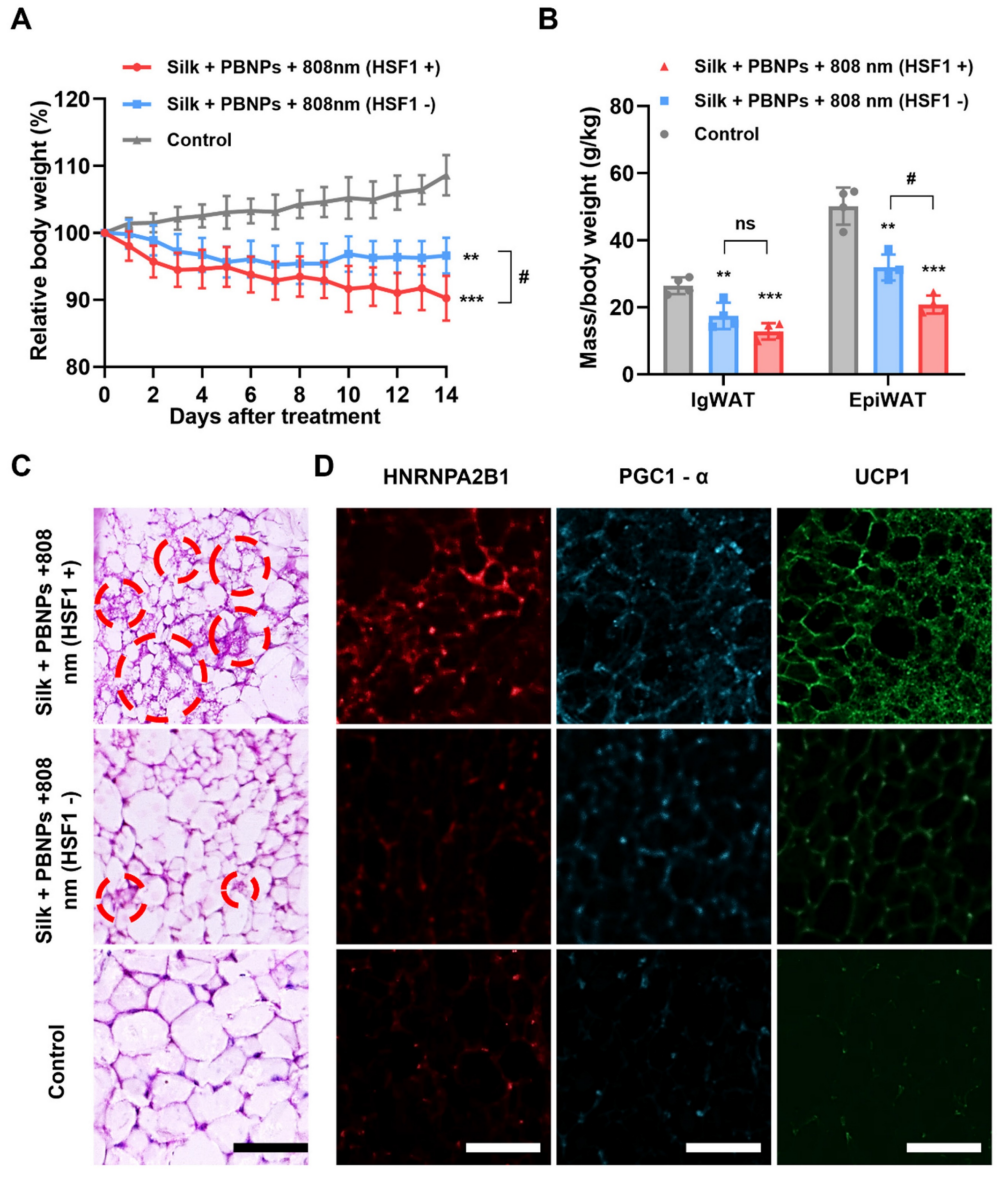
Browning of WAT. **(A)** Relative body weight changes over the course of different treatments. **(B)** Relative fat masses of IgWAT and EpiWAT. One-way ANOVA: *P < 0.05, **P < 0.01, ***P < 0.001 versus control, #P< 0.05 between indicated groups. **(C)** H&E staining images of IgWAT. Red circle indicates a representative brown-like adipocyte with multilocular morphology. **(D)** HNRNPA2B1-labeled (red), PGC1-α-labeled (blue) and UCP1-labeled (green) immunofluorescence images of IgWATs after different treatments. Scale bar = 100 μm.

We recently demonstrated that TRPV1 non-selective cation channel plays a critical role in mPTT-induced browning 13. Consistently, the activation of TRPV1 in 3T3-L1 adipocytes was evidenced by the sharp increase of intracellular calcium levels upon mPTT (**Figure S16**). On the other hand, another group recently proposed that mPTT induces browning by activating heat shock factor 1 (HSF1) because it promotes the expression of an RNA-binding protein HNRNPA2B1 (A2B1) which in turn stabilizes the transcription of two crucial proteins for thermogenesis, i.e., PGC1-α and UCP1 14. Indeed, we also observed that A2B1, PGC1-α and UCP1 were significantly augmented in the mouse IgWAT after mPTT treatment (**Figure 4F and Figure S17**). Additionally, the treatment significantly enhanced the expression of brown adipogenesis biomarkers in the IgWAT, including PRDM16, PPARγ, PGC1-α, and UCP1 (**Figure S18**). These findings suggest that the browning process is stimulated through both TRPV1 and HSF1 signaling pathways. As depicted in **Figure 5A and 5B**, intraperitoneal (IP) injection of DTHIB (an inhibitor that degrades nuclear HSF1 27) largely reduced the effectiveness of the therapy (3.45% vs. 9.78% weight reduction; 42.65% vs. 54.56% IgWAT reduction; 46.88% vs. 66.74% EpiWAT reduction), suggesting the co-involvement of HSF1 and TRPV1 signaling to create synergy (**Figure 1**). In addition, H&E staining revealed that the IgWAT of HSF1- mice exhibited fewer multilocular brown-like adipocytes compared to HSF1+ mice (**Figure 5C**), attributable to suppressed expression of A2B1, PGC1-α and UCP1 (**Figure 5D and Figure S19**).

### Obesity Associated Metabolic Disorders Relieved by mPTT-NCT

**Figure 6:**
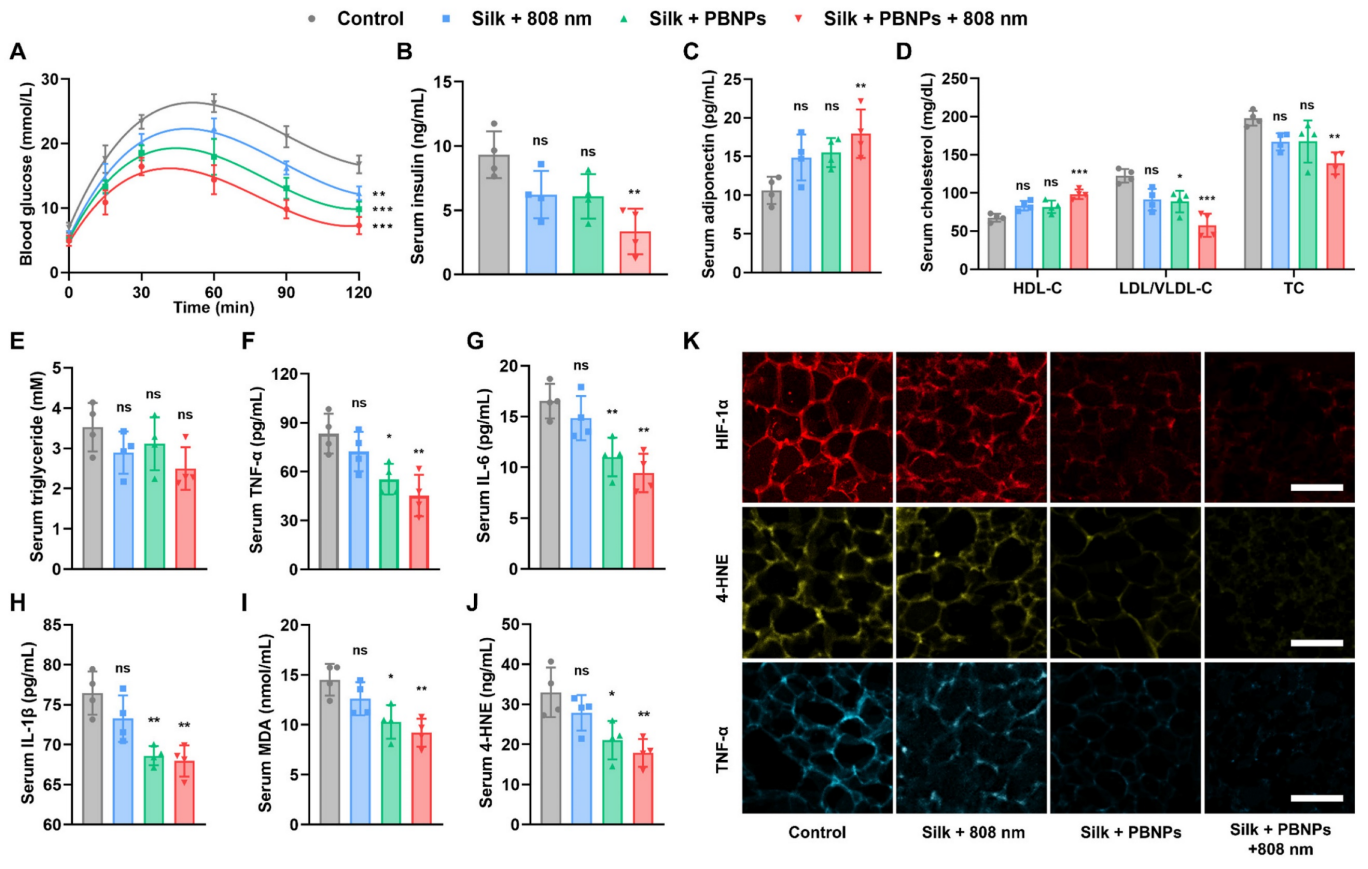
Alleviation of obesity associated metabolic disorders. **(A)** Glucose tolerance test on day 12: change of blood glucose level after glucose solution (1 g/kg in PBS) was intraperitoneally injected. **(B-J)** Serum levels of insulin, adiponectin, high-density lipoproteins (HDL), low-density lipoprotein/very low-density lipoproteins (LDL/VLDL), total cholesterol (TC), triglyceride, TNF-α, IL-6, IL-1β, MDA and 4-HNE on Day 14. **(K)** HIF-1α-labeled (red), 4-HNE-labeled (yellow) and TNF-α-labeled (blue) immunofluorescence images of IgWATs after different treatments. Scale bar = 100 μm. Data represents mean ± s.d. (n = 4). One-way ANOVA: *P < 0.05, **P < 0.01, ***P < 0.001 versus control.

The obese mice have hyperlipidemia, systemic inflammation as indicated by high serum TNF-α level, type 2 diabetes as indicated by hyperglycemia and hyperinsulinemia (**Figure 6**). To assess the effectiveness of the treatments in improving systemic metabolic health, the serum insulin level and glucose tolerance test of the treated obese mice were compared with that of healthy mice on a normal diet. It was observed that diabetes can be partially relieved by nanocatalytic therapy (without laser irradiation) and totally removed by mPTT-NCT (**Figure 6A, 6B and Figure S20**). Desirably, the lipolysis of adipocytes stimulated by the treatment did not cause an obvious increase in serum level of free fatty acid (FFA). The combined therapy led to a significant improvement in obesity-associated insulin resistance (insulin level × FFA level) compared to the control mice (**Figure S21**).

Adiponectin, which is an adipokine secreted by adipocytes, exerts insulin-sensitizing effects 28-30. As shown in **Figure 6C**, serum adiponectin level significantly increased after mPTT-NCT. Also desirably, the serum level of high-density lipoproteins (HDLs which transport lipids from peripheral tissues to the liver for excretion from the body) was increased, while the serum level of low-density lipoprotein/very low-density lipoproteins (LDLs/VLDLs which transport lipids to peripheral tissues) and total cholesterol were lowered (**Figure 6D**). But mPTT-NCT did not significantly lower the serum triglyceride level (**Figure 6E**). In mPTT-NCT treated mice, the macrophage infiltration in IgWAT was significantly less compared to the control mice, suggesting the anti-inflammatory effect of mPTT-NCT (**Figure S22**). Furthermore, the combined therapy significantly reduced the levels of pro-inflammatory cytokines (TNF-α, IL-6, IL-1β) and oxidative stress markers (malondialdehyde - MDA, 4-Hydroxynonenal - 4-HNE) in serum (**Figure 6F-J**). These findings were consistent with the suppression of HIF-1α (hypoxia indicator), 4-HNE, and TNF-α expression in IgWAT of mice treated by mPTT-NCT (**Figure 6K and Figure S23**). In comparison, the effects of nanocatalytic therapy alone (without laser irradiation) are not as significant as the combined therapy, highlighting the importance of the synergy between mPTT and NCT.

### Biosafety Studies

**Figure 7:**
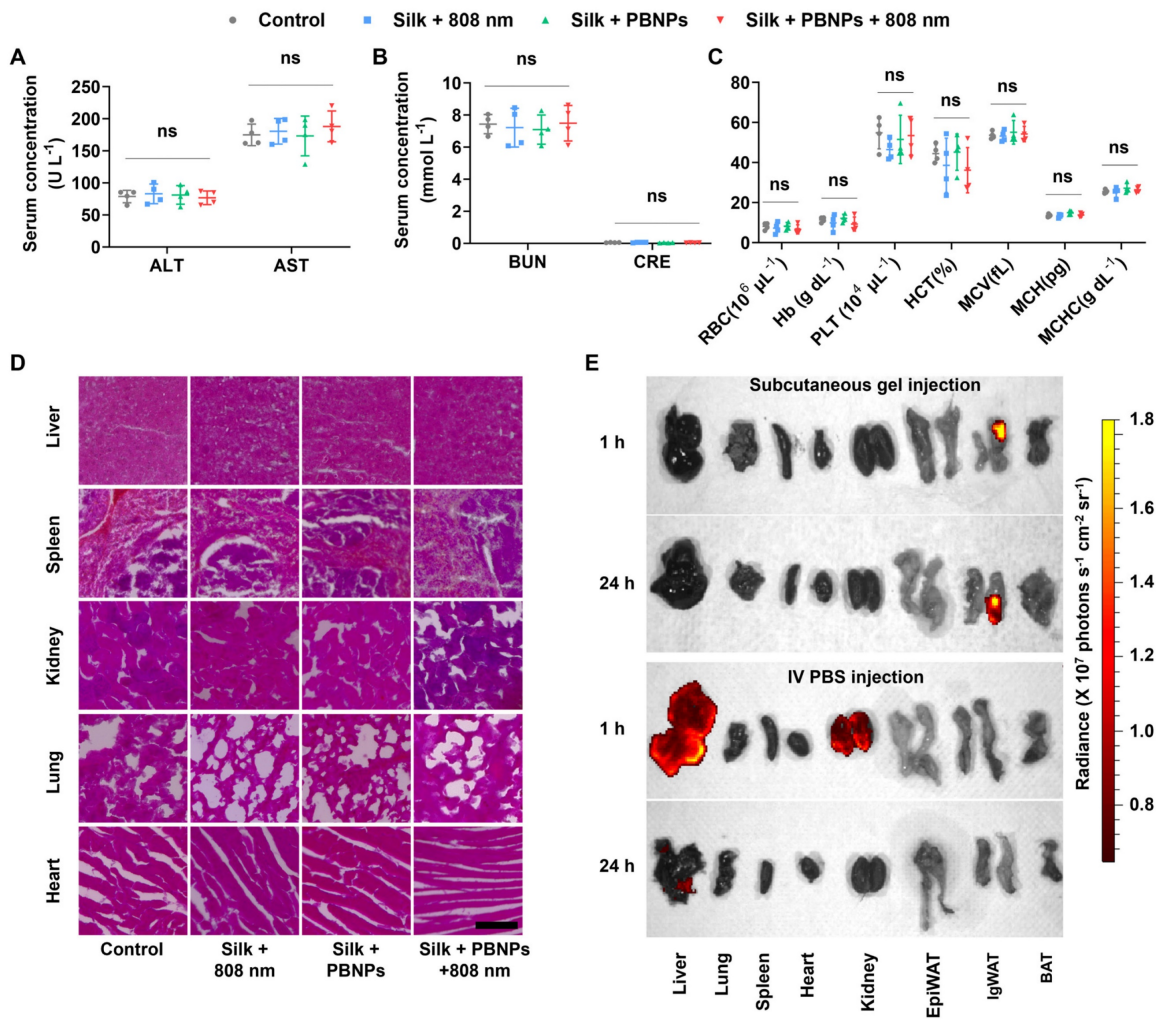
Biosafety and *in vivo* biodistribution of PBNPs. **(A)** Serum levels of liver function indicators: alanine transaminase (ALT), aspartate transaminase (AST). **(B)** Serum levels of kidney function indicators: blood urea nitrogen (BUN), creatinine (CRE). **(C)** Hematologic analysis: red blood cell (RBC), hemoglobin (Hb), platelet (PLT), hematocrit (HCT); mean corpuscular volume (MCV), mean corpuscular hemoglobin (MCH) and mean corpuscular hemoglobin concentration (MCHC) Data represents mean ± s.d. (n = 4) One-way ANOVA: ns = not significant; between indicated groups. **(D)** H&E staining images of major organs. Scale bar = 100 µm. **(E)** *In vivo* biodistribution of Cy5-PBNPs in major organs and fat tissues (EpiWAT, IgWAT, brown adipose tissue - BAT) of mice, 1 or 24 h after subcutaneous injection of silk gel containing Cy5-PBNPs (0.15 mg/kg) onto one side of the inguinal region or IV injection of Cy5-PBNPs in PBS (0.15 mg/kg).

As shown in **Figure 7A and 7B**, all the treatments did not significantly alter the blood biomarkers related to liver function (alanine transaminase - ALT, aspartate transaminase - AST) and kidney function (blood urea nitrogen - BUN, creatinine - CRE). Also, hematologic parameters (red blood cell - RBC, hemoglobin - Hb, platelet - PLT, hematocrit - HCT, mean corpuscular volume - MCV, mean corpuscular hemoglobin - MCH, mean corpuscular hemoglobin concentration - MCHC) remained normal (**Figure 7C**). Additionally, no signs of damage, congestion, or hemorrhage were observed in the liver, kidneys, spleen, lungs, or heart (**Figure 7D**). These observations indicate that mPTT-NCT is safe and promising for clinical application.

The excellent biosafety is attributable to not only the biocompatibility of PBNPs and silk hydrogel but also the localized transdermal delivery directly into subcutaneous WAT. The biodistribution of fluorescent Cy5-conjugated PBNPs (Cy5-PBNPs) was observed after subcutaneously injecting Cy5-PBNPs-loaded silk gel into mouse IgWAT or systemic administration through intravenous (IV) injection (**Figure 7E**). After subcutaneous injection, nearly all and ~81% of Cy5-PBNPs remained in the IgWAT after 1 and 24h respectively. This indicates that the silk gel can effectively prolong the retention of Cy5-PBNPs at the injection site, allowing for sustained therapeutic effect. In contrast, IV injected Cy5-PBNPs mainly accumulated in the kidney and liver, with minor presence in the heart, lung, and spleen.

## Discussion

Based on the comprehensive experimental results, the working mechanism involving complex interconnected signaling pathways is proposed to explain the beneficial remodeling of the diseased obese sWAT and the systemic effects induced by the synergized mPTT-NCT (**Figure 1**). Specifically, the PBNP-enabled photothermal effect simultaneously activates TRPV1 and HSF1 signaling pathways. Ca^2+^ flow through TRPV1 channels and heterogeneous nuclear ribonucleoprotein A2B1 activated by HSF1 stimulate the expression of PGC1-α, which in turn leads to expression of thermogenic protein UCP1 and increased biogenesis of mitochondria (i.e., transformation of white adipocytes to thermogenic brown-like adipocytes) 14. In parallel, Ca^2+^-induced expression of peroxisome proliferator-activated receptor γ (PPARγ) stimulates the expression of hormone-sensitive lipase (HSL) to promote lipolysis 13. Consequently released free fatty acids (FFAs) are then consumed as the thermogenic fuel by brown-like adipocytes as evidenced by body temperature increase.

Adipose tissue is a major endocrine organ, which secretes a variety of signaling molecules called adipokines (e.g., adiponectin) to regulate whole-body energy metabolism. In obese fat, oxidative stress, hypoxia and inflammation form vicious positive couplings (**Figure 1**). These pathological conditions lead to systemic metabolic disorders because of the altered secretion of adipokines and release of pro-inflammatory factors and deleterious molecules (e.g., free fatty acids). The ROS scavenging and O_2_ producing capabilities of PBNP suppress these pathological conditions and break their coupling. Consequently, the release of inflammatory factors is decreased while the secretion of adiponectin is increased. Through PPARγ activation, TRPV1-enabled Ca^2+^ flow also stimulates adiponectin secretion 31, 32. Adiponectin enhances insulin sensitivity, hepatic synthesis of lipid transporters (HDL) and FFA oxidation in peripheral tissues and liver, thereby leading to reduction of insulin resistance and size of adipocytes in visceral fat because of enhanced lipolysis.

Our approach can be translated for human use. For example, the following treatment protocol may be envisioned. Hydrogel (1 mL) containing PBNPs (50 µg) can be self-injected into belly fat at 8 different sites using an insulin needle once a week. Then a hand-held NIR laser can be used daily to apply irradiation for 5 min at each site. Hyperthermia can be generated in ~1 cm diameter area at each injection site. For this protocol, the corresponding iron dosage is only 0.18 mg/week, which is significantly below the safe limit for human iron intake (45 mg/day) 33.

## Conclusions

In summary, we proposed a new strategy to beneficially remodel obese subcutaneous white adipose tissue (sWAT) through the mild-photothermal and nanocatalytic therapy which is enabled by silk fibroin hydrogel based transdermal delivery of Prussian blue nanoparticles. The synergized therapy not only leads to significant mass reduction of the targeted sWAT but also improves the whole-body metabolism as evidenced by total relief of type 2 diabetes, as well as large reduction of visceral fat, hyperlipidemia, systemic oxidative stress, and systemic inflammation. Our experiments carefully revealed that the photothermal effect stimulates browning through both TRPV1 and HSF1 signaling pathways, and the ROS scavenging and O_2_-producing ability of PBNPs breaks the vicious couplings between oxidative stress, hypoxia and inflammation. Both photothermal and nanocatalytic therapies stimulate the secretion of adiponectin into the circulation which plays the key role in improving systemic metabolism. This study provides new insights to inspire novel strategies to combat obesity and associated diseases.

## Supplementary Material

Supplementary figures.
