# Supplementary material for: Mild-photothermal and nanocatalytic therapy for obesity and associated diseases
Source: Theranostics. 2024 Sep 3;14(14):5608–20. doi: 10.7150/thno.99948 (PMC11413782; doi:10.7150/thno.99948)
Supplement: Supplementary file 1 — Supplementary figures. [file thnov14p5608s1.pdf]

Supplementary Material for

Mild-photothermal and nanocatalytic therapy for obesity and associated diseases

Lewen Zheng [1], Aung Than [1], Ping Zan [1], Dongsheng Li [2], Zheye Zhang [1], Melvin Khee Shing Leow [3,4], Peng Chen [1,4,5,*]

[1] *School of Chemistry, Chemical Engineering and Biotechnology, Nanyang Technological University, 637457, Singapore.*

[2] *College of Materials and Chemical Engineering, Key Laboratory of Inorganic Nonmetallic Crystalline and Energy Conversion Materials, China Three Gorges University, Yichang, 443002, P. R. China.*

[3] *Department of Endocrinology, Tan Tock Seng Hospital, 308433, Singapore.*

[4] *Lee Kong Chian School of Medicine, Institute for Digital Molecular Analytics and Science, Nanyang Technological University, 636921, Singapore.*

[5] *Skin Research Institute of Singapore, 308232, Singapore.*

*Corresponding author. Email: chenpeng@ntu.edu.sg

Methods

Cell differentiation

3T3-L1 pre-adipocytes were chemically induced to differentiate into mature adipocytes with differentiation medium (DMEM supplemented with 10% FBS, 0.5 mM 3-isobutyl-1-methylxanthine, 1 μM dexamethasone, 2 μM rosiglitazone and 1 μg/ml insulin). After 72 h, the cells were cultured with DMEM medium supplemented with 10% FBS and 1 μg/ml insulin, which was replenished every two days in the following 10 days before conducting the experiments.

Cytotoxicity test

3T3-L1 adipocytes were incubated with DMEM containing different concentrations of PBNPs (0-250 μg/mL). After 24 h, the adipocytes were incubated with fresh DMEM medium supplemented with 10% Cell Counting Kit 8 (CCK8) for 2 h at 37 °C. The viability was then determined by measuring absorbance intensity at 460 nm.

Cellular oxidative stress test

3T3-L1 adipocytes were incubated with DMEM (Untreated), or DMEM containing 100 μg/mL PBNP (PBNP), or DMEM containing 500 μM H_2O_2 (H_2O_2), or DMEM containing 500 μM H_2O_2 and 100 μg/mL PBNP (H_2O_2 + PBNP) at 37 °C. After 3 h, the adipocytes were incubated with fresh DMEM medium containing 20 μM dichlorodihydrofluorescein diacetate (DCFH-DA) for 1 h at 37 °C. The Cellular oxidative stress level was measured under a confocal microscope (Zeiss LSM 800).

In Vitro Experiments on Adipocytes

3T3-L1 adipocytes were cultured in DMEM medium and either left untreated (control) or treated with PBNP-gel (20 μL containing 50 μg/mL PBNPs) for 24 hours. Subsequently, the adipocytes underwent irradiation with an NIR laser (808 nm, 0.4 W/cm^2) for 5 minutes. After 12 hours post-NIR irradiation, cellular malondialdehyde (MDA) levels were assessed using a colorimetric quantification kit. Intracellular lipid droplets were visualized by staining with Oil Red O (Sigma-Aldrich) and examined under a microscope. Twenty-four hours following NIR irradiation, adipocytes were labeled with specific primary antibodies (Thermo Fisher Scientific) and then incubated with fluorescence dye-conjugated secondary antibodies (Thermo Fisher Scientific). Immunofluorescence staining was observed using a confocal laser scanning microscope (CLSM) (LSM800, Carl Zeiss).

Fig.s

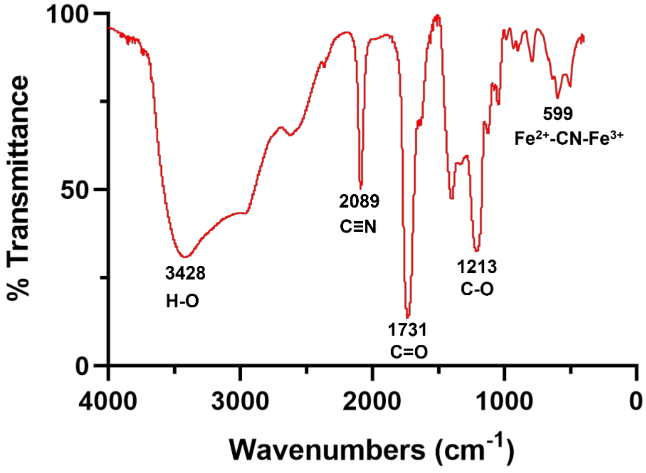

Figure S1. FTIR spectrum of PBNP.

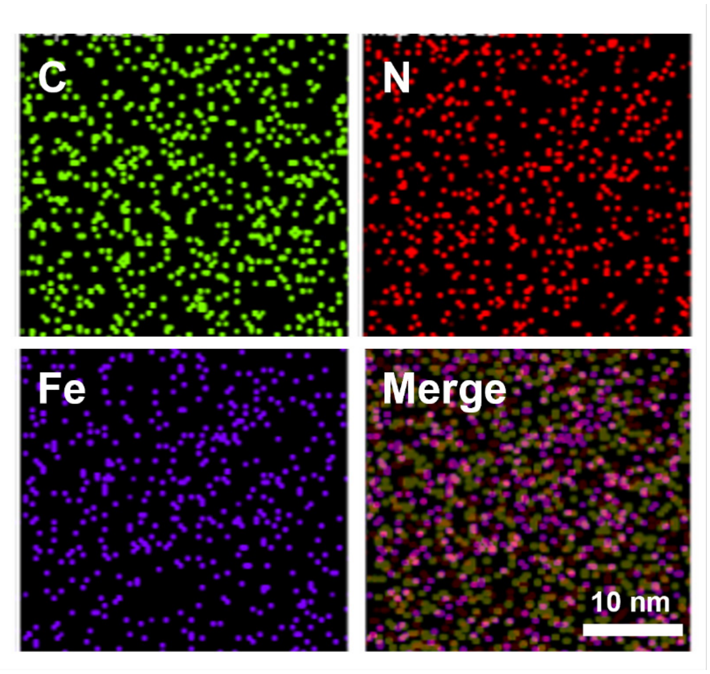

Figure S2. Energy Dispersive X-ray elemental mapping images of PBNPs.

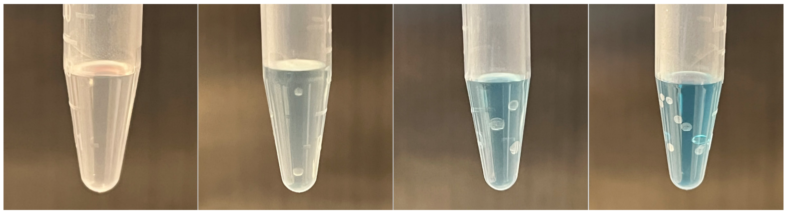

Figure S3. The photographs of 0, 25, 50 and 100 μg/mL PBNP after being incubated in 50 mM H_2O_2 containing PBS for 20 minutes.

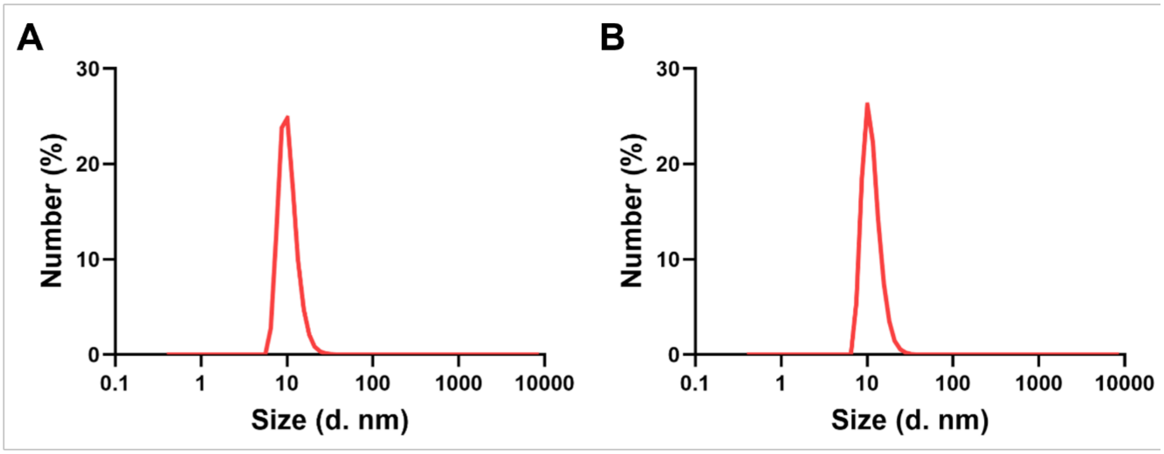

Figure S4. DLS measurement of PBNPs (**A)** before and (**B)** after SOD activity test.

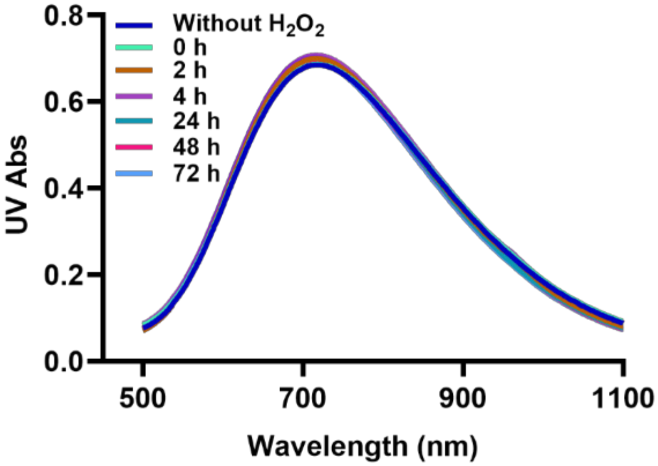

Figure S5. The UV absorbance of PBNP being incubated in 50 mM H_2O_2 containing PBS for different time durations.

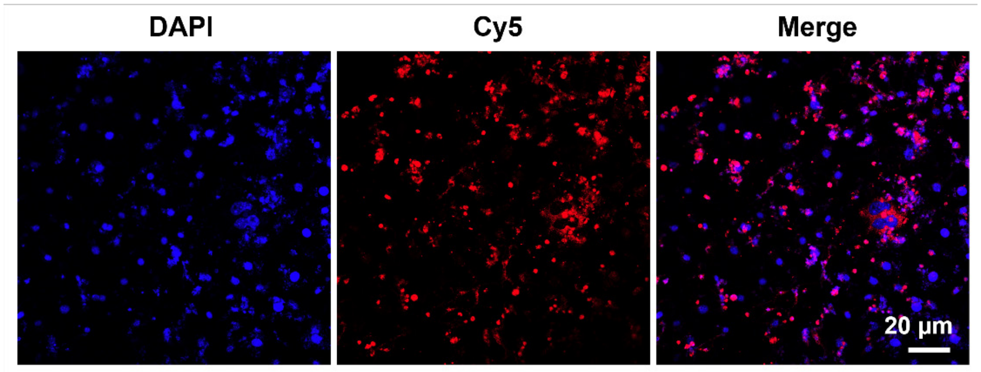

Figure S6. Fluorescence images showing cellular uptake of Cy5-PBNPs by 3T3-L1 adipocytes after 2 h of incubation.

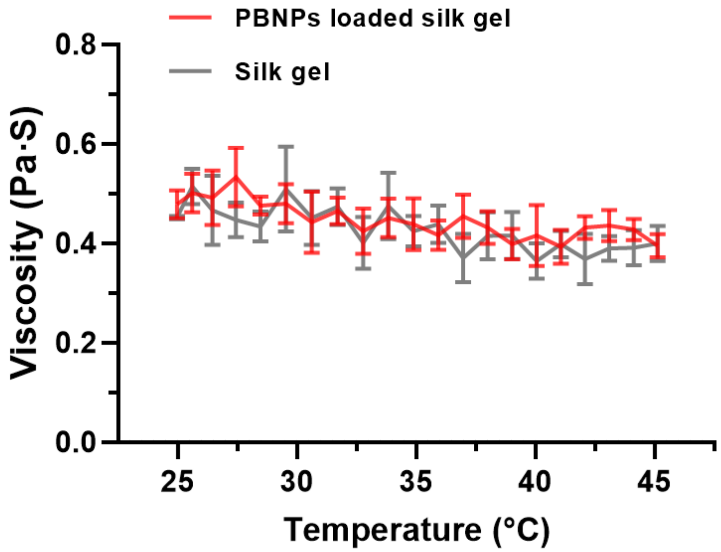

Figure S7. Viscosity analysis of PBNP-gel by gradually increasing temperature from 25 to 45 ℃ at a constant shear rate (1 s^{-1}). Data represents mean ± s.d. (n = 3).

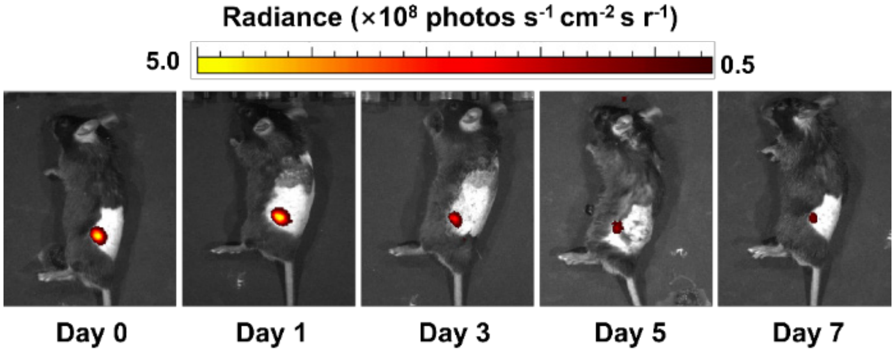

Figure S8. In vivo fluorescence images of Cy5-PBNPs on different days after subcutaneous injection of Cy5-PBNP incorporated silk gel.

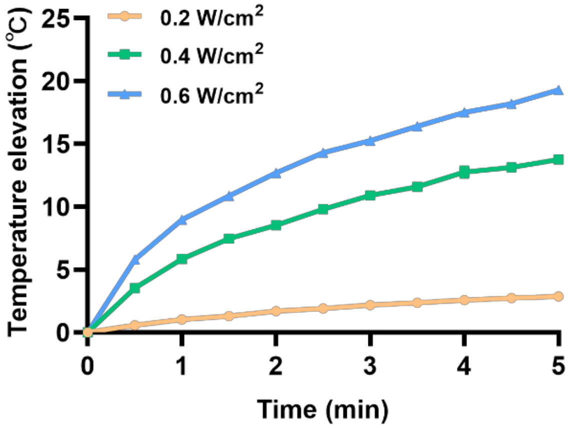

Figure S9. Real-time temperature changes of silk hydrogel containing 50 μg/mL PBNPs upon 808 nm NIR irradiation with different power densities.

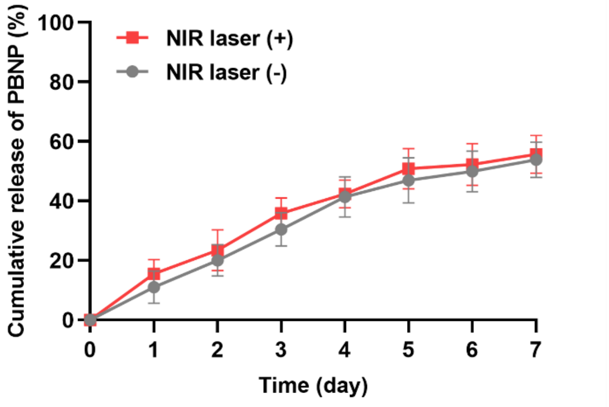

Figure S10. Photothermal effect on the release of PBNPs from silk gel in vitro.

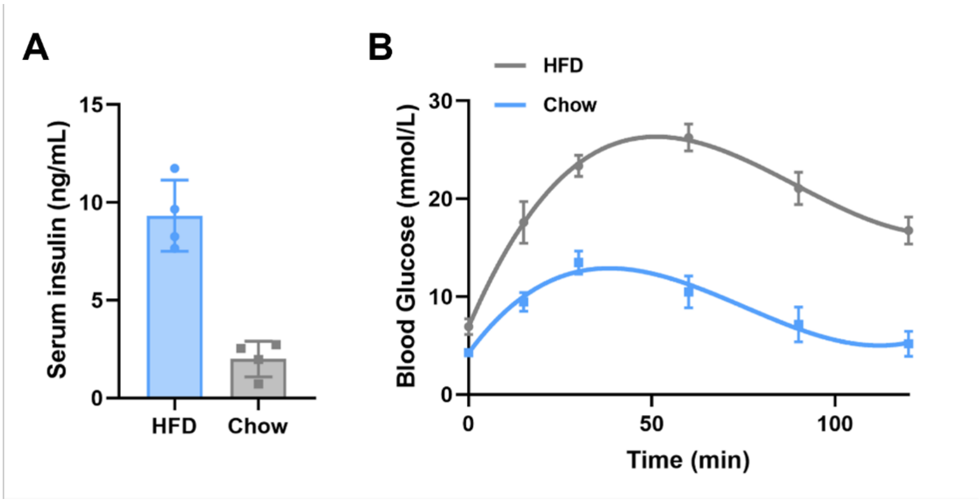

Figure S11. (A) Serum insulin levels of high-fat diet (HFD) fed mice and standard chow (Chow) fed mice. **(B)** Change of blood glucose level of HFD-fed mice and Chow-fed mice after glucose solution (1 g/kg in PBS) was intraperitoneally injected at t = 0 min. Data represents mean ± s.d. (n = 4).

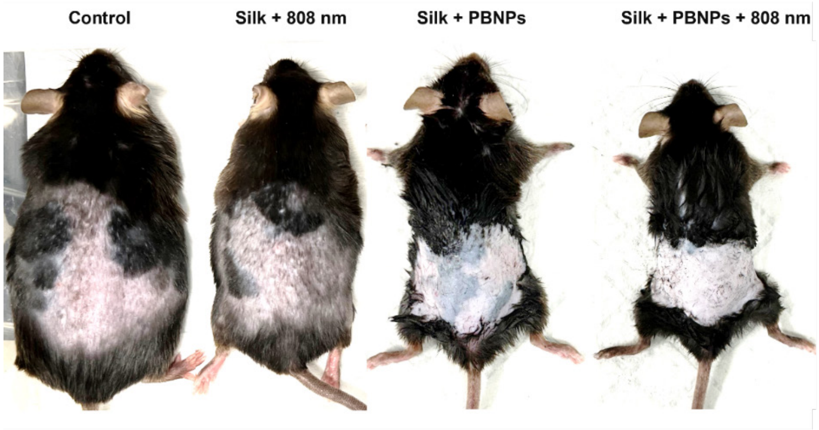

Figure S12. The photographs of mice from different groups on day 14.

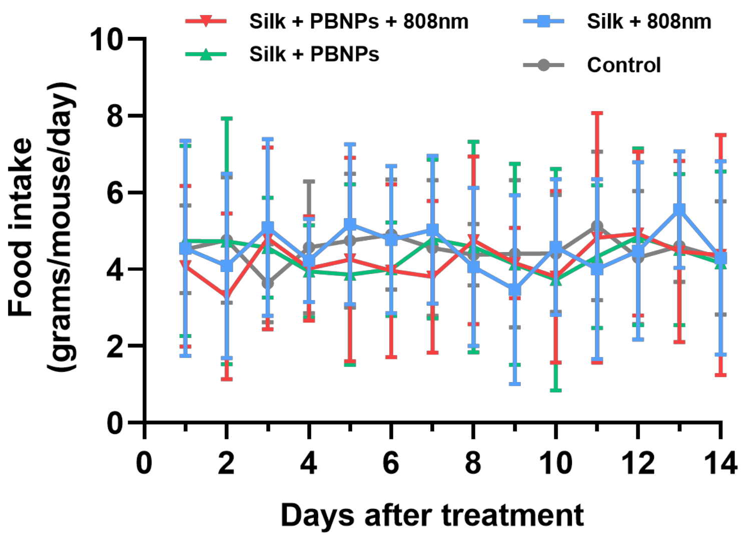

Figure S13. Food intake of mice from four groups. Data represents mean ± s.d. (n = 3).

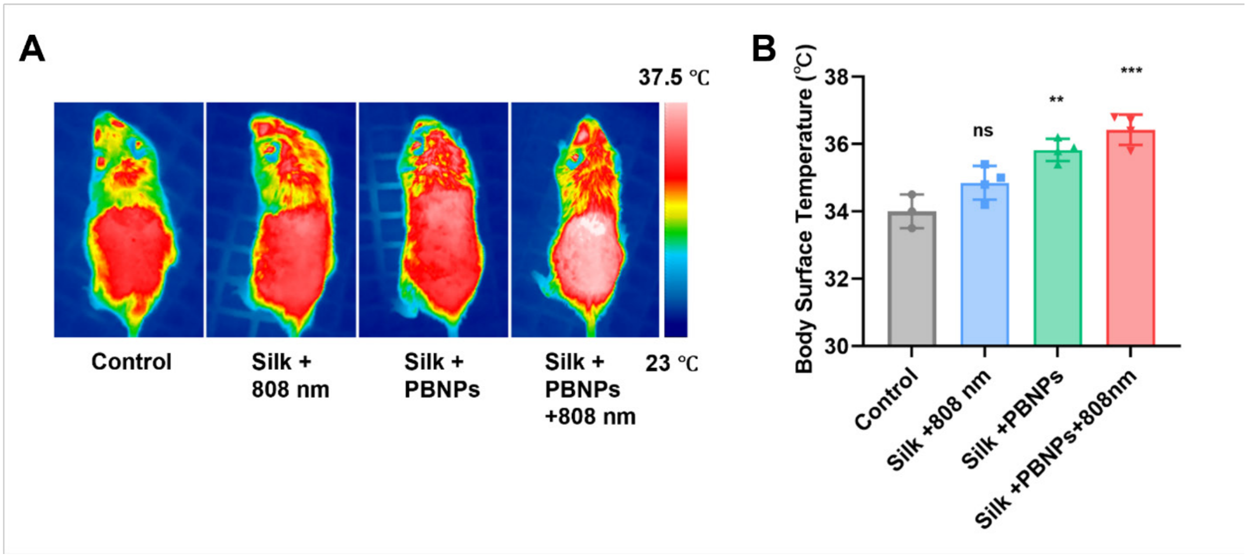

Figure S14. Thermal images **(A)** and average body surface temperature of differently treated mice **(B)** on day 14. Data represents mean ± s.d. (n = 4). One-way ANOVA: ns = not significant; **P < 0.01, ***P < 0.001 versus control.

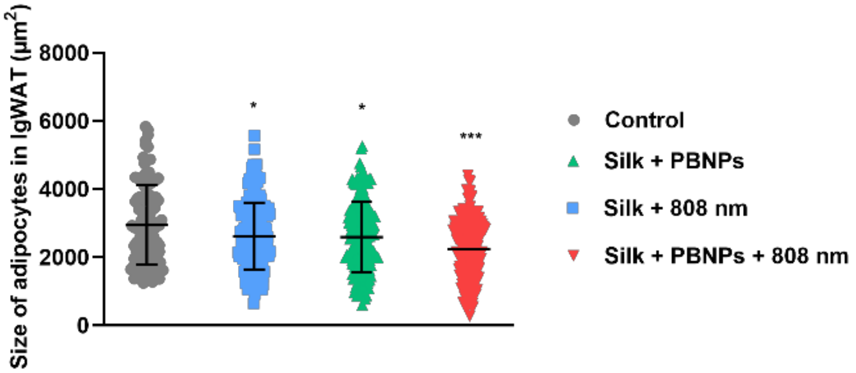

Figure S15. Adipocyte size from the experiments shown in **Figure3e** (200 data points for each group with mean and s.d. indicated). One-way ANOVA: ns = not significant; *P < 0.05, ***P < 0.001 versus control.

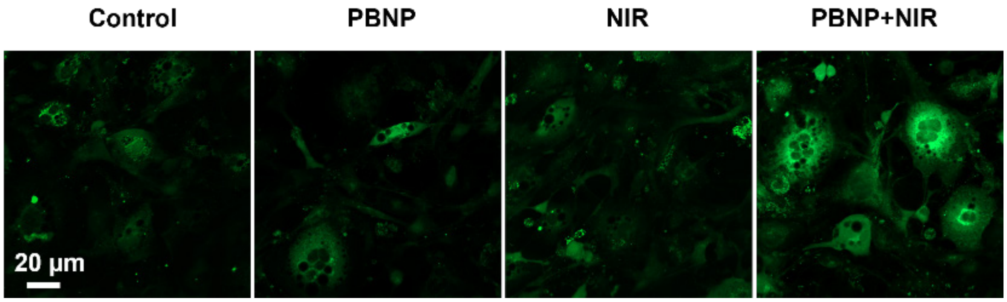

Figure S16. Fluorescence images of intracellular calcium in 3T3-L1 adipocytes.

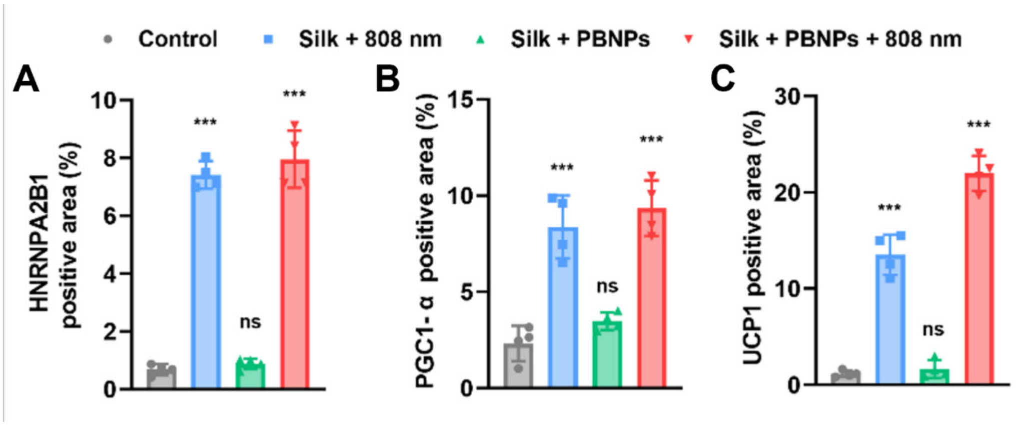

Figure S17. HNRNPA2B1 **(A)**, PGC1-α **(B)**, and UCP1 **(C)** positive area (%) from the experiments shown in **Figure 4f**. Data represents mean ± s.d. (n = 4). One-way ANOVA: ns = not significant; ***P < 0.001 versus control.

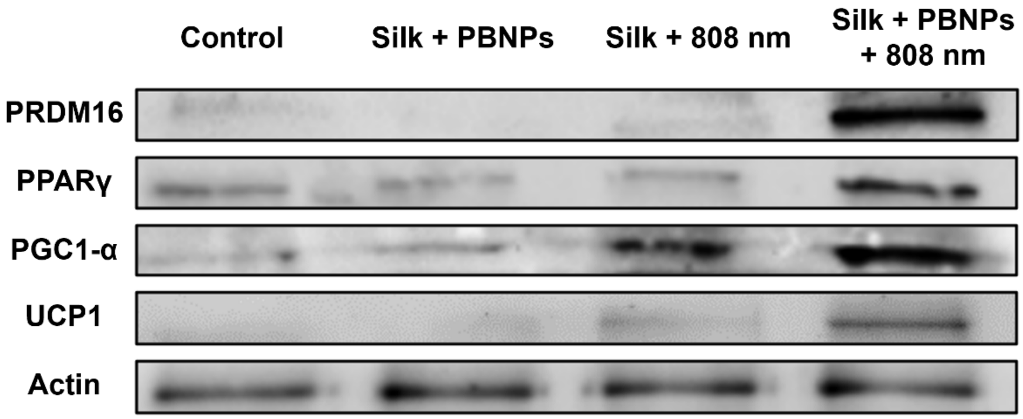

Figure S18. Immunoblots of PRDM16, PPARγ, PGC1-α, UCP1 and actin in IgWATs.

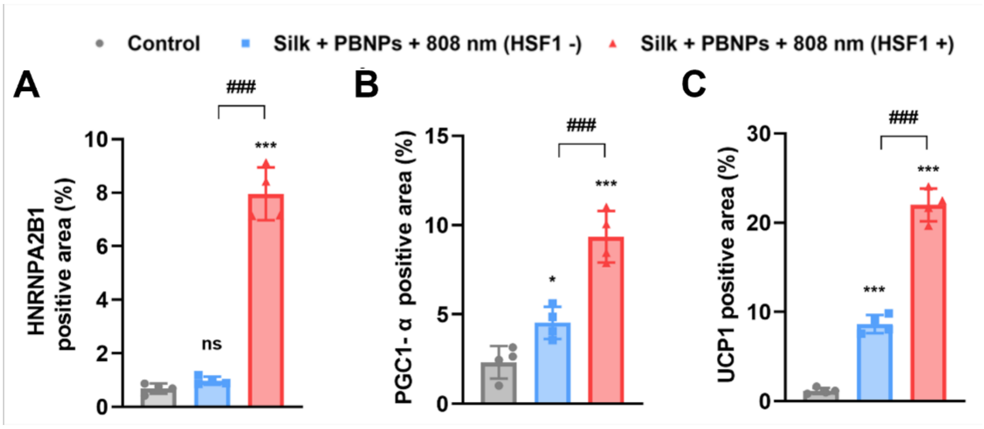

Figure S19. HNRNPA2B1 **(A)**, PGC1-α **(B)**, and UCP1 **(C)** positive area (%) from the experiments shown in **Figure 5d**. Data represents mean ± s.d. (n = 4). One-way ANOVA: ns = not significant; *P < 0.05, ***P < 0.001 versus control, ###P < 0.001 between indicated groups.

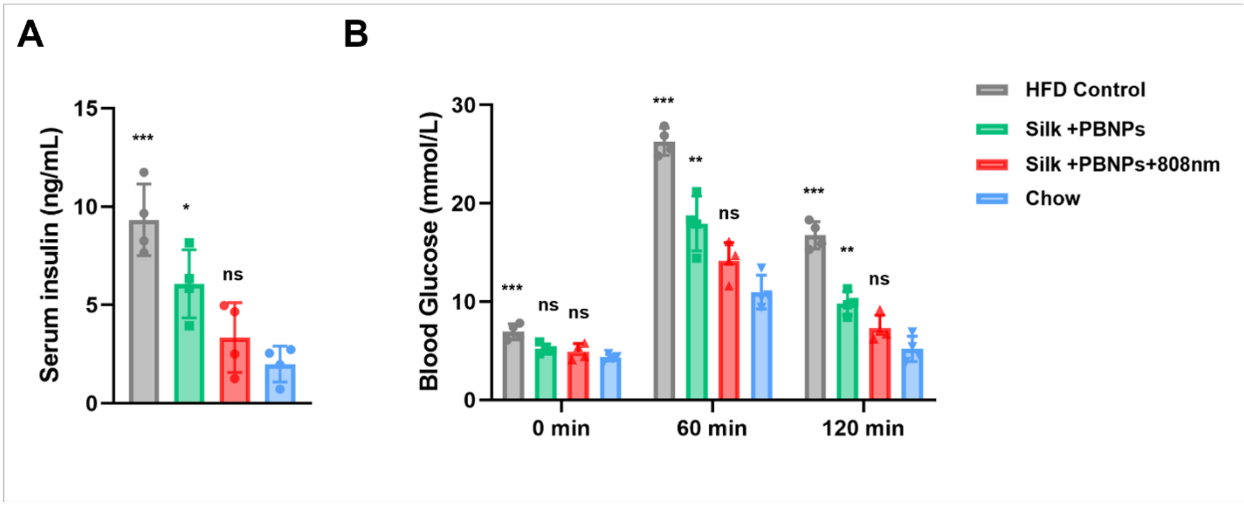

Figure S20. (A) Serum insulin levels of high-fat diet (HFD) fed obese mice, silk + PBNPs treated obese mice, silk + PBNPs + 808 nm treated obese mice, standard chow (Chow) fed healthy mice. **(B)** Blood glucose level of HFD-fed control mice, silk + PBNPs treated mice, silk + PBNPs + 808 nm treated mice and Chow-fed mice after glucose solution (1 g/kg in PBS) was intraperitoneally injected at t = 0 min. Data represents mean ± s.d. (n = 4). ns = not significant; $^{**}P < 0.01$, $^{***}P < 0.001$ versus Chow.

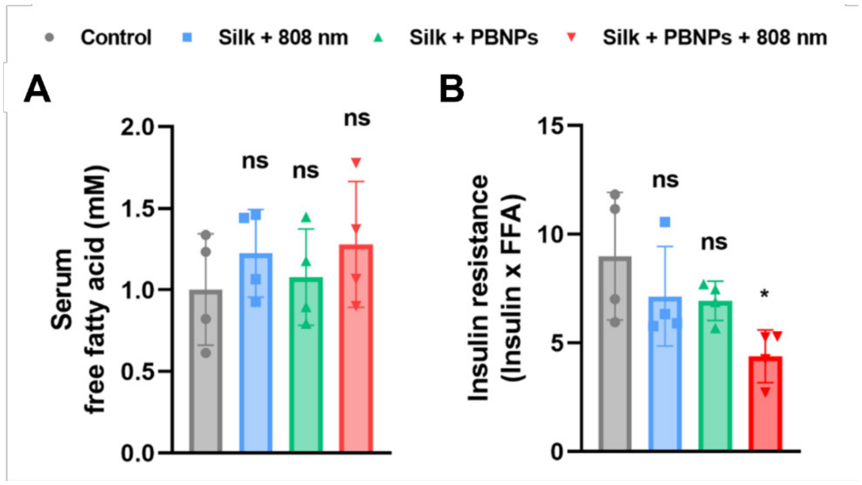

Figure S21. (A) Serum levels of free fatty acid (FFA) of differently treated mice. **(B)** Insulin resistance index. Insulin resistance was calculated as insulin level x FFA level. Data represents mean ± s.d. (n = 4). One-way ANOVA: ns = not significant, *P < 0.05 versus control.

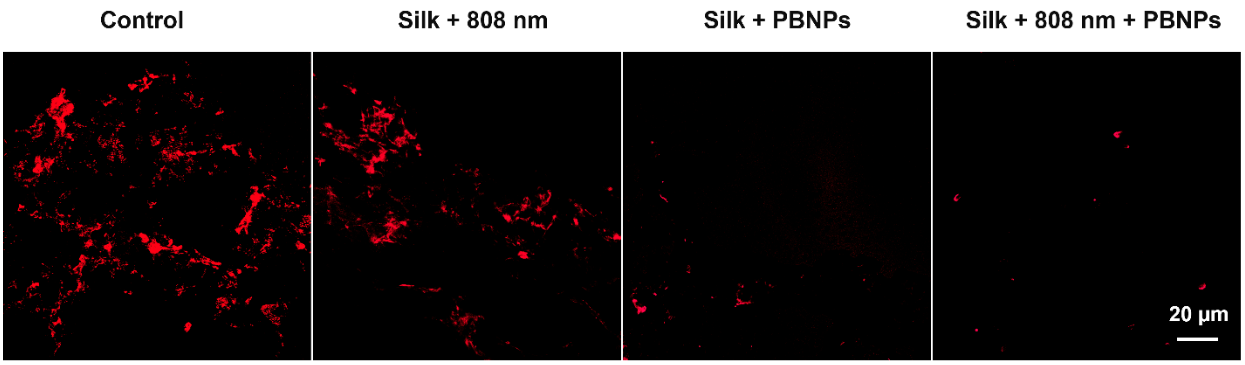

Figure S22. F4/80-labeled (red), immunofluorescence images of IgWATs after different treatments.

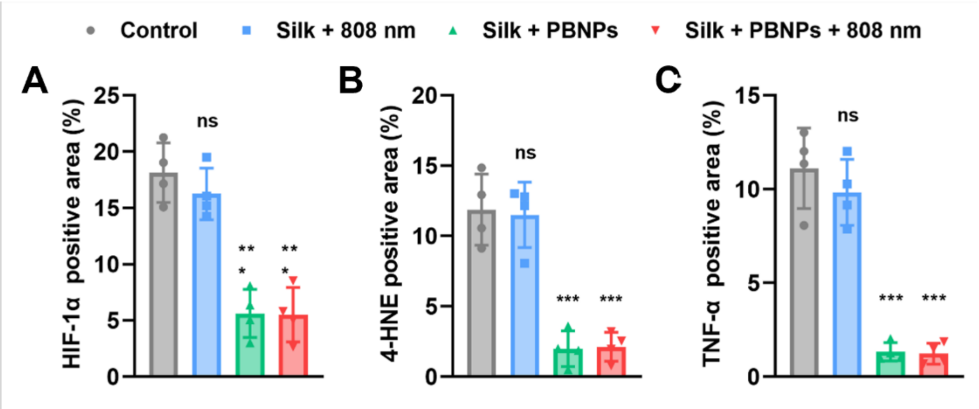

Figure S23. HIF-1α **(A)**, 4-HNE **(B)** and TNF-α **(C)** positive area (%) from the experiments shown in **Figure 6k**. Data represents mean ± s.d. (n = 4). One-way ANOVA: ns = not significant; ***P < 0.001 versus control.
